# Engaging a Community-Academic Partnership to Implement Community-Driven Solutions

**DOI:** 10.5888/pcd22.240334

**Published:** 2025-06-05

**Authors:** Abiola O. Keller, Lindsey St. Arnold Bell, Kristin Haglund

**Affiliations:** 1 College of Nursing, Marquette University, Milwaukee, Wisconsin; 2 Near West Side Partners, Milwaukee, Wisconsin

## Abstract

Community engagement is a pivotal public health tool for addressing population health challenges and advancing health equity. Community–academic partnerships that use community-engaged approaches can prioritize community strengths and ensure that resources and interventions match local needs. In 2021–2022, a community-academic partnership, guided by the principles of community engagement, collaborated with residents of Milwaukee’s Near West Side (NWS) to identify strengths and assets and prioritize actions to improve health and quality of life. To inform the development of a planned community resource center, residents were invited for group concept mapping (GCM).

GCM includes idea generation, sorting and rating, and developing cluster maps. Residents (N = 165) generated 71 unique ideas in response to the question, “To make the Near West Side a healthier community we need _____.” Residents sorted ideas into clusters based on conceptual similarity and prioritized the importance of each. Data were managed with The Concept System Global MAX Software. By using the 71 ideas, a cluster map with 9 domains best fit the data. Domains were high-quality and affordable housing, community-engaged public safety, health and wellness services, strong and inclusive neighborhoods, investments in young people, public infrastructure, sustainable businesses, alternative modes of transportation, and vibrant social spaces. Eight of the 9 domains were highly rated for importance. These domains became focus areas for our partnership’s efforts to advance health and well-being in NWS. Our work highlights the significance of projects incorporating community engagement principles within the context of a community–academic partnership to generate mutually beneficial solutions that are strength-based and aligned with partners’ priorities.

**Summary**

**What is already known on this topic?**

Community-engaged research offers a mutually beneficial approach for communities and organizations/institutions to work together to solve entrenched issues that contribute to inequities, decrease public health, and impede quality of life. Little is known about leveraging community–academic partnerships to implement change.

**What is added by this report?**

We provide an example of a community–academic project that harnessed the knowledge and expertise of residents to inform interventions to support better acceptance, uptake, and efficacy.

**What are the implications for public health practice?**

Our work highlights the significance of projects incorporating community engagement principles within the context of a community–academic partnership to generate solutions aligned with partners’ priorities.

## Community Engagement to Advance Public Health Equity

In urban centers, equity issues related to public health and quality of life are complex. Economic disadvantage is often concentrated and can adversely affect the health of residents, prevent investment and development, deter patronage of local businesses, and contribute to actual or perceived increased crime rates. Community-engaged research (CEnR) is a mutually beneficial approach for communities, organizations, and institutions to work together to create solutions that promote equity and public health and improve quality of life.

CEnR is an umbrella term for participatory-oriented research methodologies (1). In CEnR, peoples’ lived experiences are important sources of knowledge. Benefits of using CEnR methodologies in public health improvement projects include identification of more nuanced and specific etiologies of underlying problems, identification of strengths and assets of populations, creation of interventions with increased likelihood of success, and generation of results that are relevant, timely, and useful to populations.

Different methodologies can be used to conduct CEnR, but 9 principles put forth by the Clinical and Translational Science Awards Consortium Community Engagement Key Function Task Force summarize the fundamental principles of this approach (2):

1. Transparency of purpose, goals, and participation
2. Shared knowledge of history and contexts of the community
3. Relationships that cultivate trust and reciprocity
4. Respect for the self-determination of a community and of its individual members
5. Inclusive partnerships
6. Diverse and culturally centered
7. Strength and asset-based
8. Co-created with shared governance
9. Sustainable

Community–academic partnerships (ie, equitable partnerships between local communities and academic research institutions) are a key tool for successfully conducting CEnR. Community–academic partnerships can provide opportunities for collaborators to augment their impact by focusing resources and increasing capacity (3). Although literature describing examples of, and factors related to, establishing and sustaining community–academic partnerships exist (4–8), literature documenting how to leverage such partnerships to implement community-driven solutions is sparse. In this article, we present an example of a community–academic partnership working to improve health and quality of life in a community. Our partnership enacted principles of community engagement (2) to engage community members in assessing strengths and assets, prioritizing actions for meaningful change, and elucidating perspectives on how to advance the health of the community.

## The Community and Partners

Milwaukee’s Near West Side (NWS), a “neighborhood of neighborhoods,” comprises 7 unique neighborhoods and is home to approximately 29,000 residents. This area was once known for thriving commercial corridors, strong connections among residents, and major employers. However, in a 20-year period, beginning in the 1970s, many large organizations, including hospitals, a medical college, and a university, left NWS, with an accompanying loss of family-sustaining jobs. With these changes, NWS lost its identity as a thriving commercial and residential corridor and instead became the hub of a city known for being among the nation’s most impoverished, incarcerated, and racially segregated (9). The median household income in NWS is $18,686, which means that 42% of its children and 46% of its families live in poverty compared with 24% of families overall who live in poverty in Milwaukee (10). The unemployment rate in NWS neighborhoods ranges from 7.3% to 14.4%, compared with 6.8% for Milwaukee overall (10).

To revitalize NWS and sustain thriving business and residential corridors, Near West Side Partners (NWSP) was founded in 2014 through the support of 5 institutions: Aurora Health Care (now Advocate Aurora Health), Harley-Davidson, Marquette University, MillerCoors (now MolsonCoors Beverage Company), and Potawatomi Business Development Corporation (now Potawatomi Ventures). As a nonprofit organization, NWSP’s goal is to make NWS a great place to live, work, play, and stay. In 2021, NWSP embarked on an initiative to establish a community resource center in NWS. It was imperative that residents of NWS be included in planning the space, amenities, and services for the resource center. As such, NWSP partnered with researchers in Marquette University’s College of Nursing to design and implement a project that would use the principles of community engagement to identify and prioritize community-driven solutions for a healthier community.

Respect for the self-determination of a community and of its individual members is critical for the sustained success of any improvement initiative (2). Self-determination is supported when community members are included in planning and implementing strategies and evaluating outcomes. As such, Group Concept Mapping (GCM) was selected as an ideal methodologic approach for this work because it provides a structured, collaborative process for generating a model of how members of the community conceptualize a healthy community (11). GCM is a way to promote the self-determination of a community and to systematically gather ideas from many people and organize those ideas into actionable priorities. Previous studies have used GCM to create frameworks (12), prioritize strategies (13), inform health-related research (14–16), and plan and evaluate programs (17).

## The Group Concept Mapping Process

**Figure 1:**
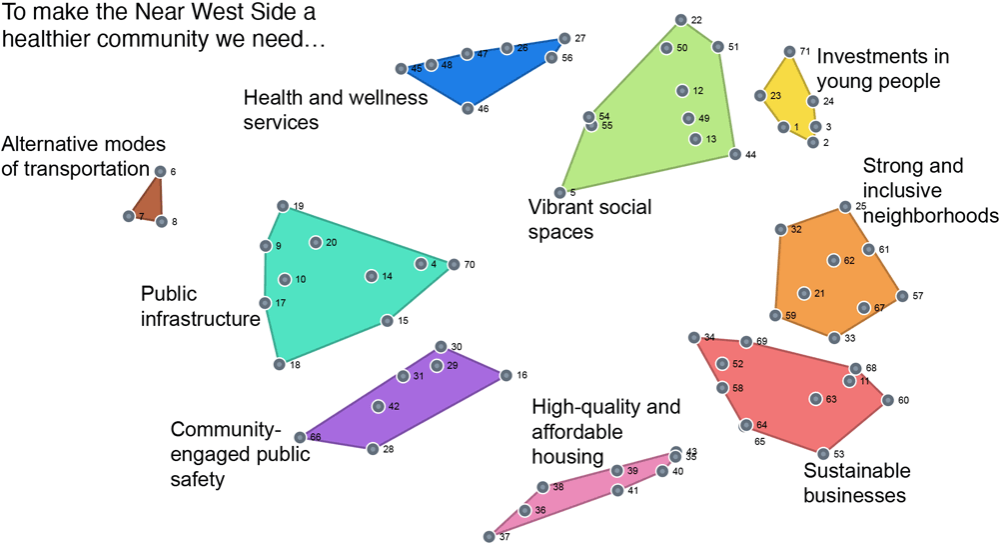
Group concept mapping cluster map showing solutions within 9 domains. The map displays ideas for improving community health and quality of life contained in each domain. The smaller in size the cluster, the greater the interrelationship between ideas within the cluster.

The GCM process consisted of 5 steps: 1) preparation, 2) idea generation, 3) sorting and rating, 4) creating maps, and 5) interpreting maps (Figure 1) (18). One author (A.O.K.) trained as a concept-mapping facilitator and oversaw each session. In 2021, data were collected from June through November. There were 165 participants for idea generation and 138 for sorting and rating. Of the sorting and rating participants, 59% were female, 77% African American, 54% aged younger than 45 years, and 93% renting their current home. Participants represented all 7 neighborhoods. All participants in idea generation and about two-thirds of participants in sorting and rating were residents of NWS. The project was reviewed by Marquette University’s institutional review board, which determined the study was not human subjects research because the intent was not to create generalizable knowledge. The study was conducted in English only.

### GCM step 1

**Preparation.** The intent of our GCM project was to gather actionable ideas from community members. A focus prompt is the question stem that participants respond to when brainstorming ideas. For our project, we aimed to devise a focus prompt that would elicit strength-based responses. We pilot tested 2 focus prompts: A, “To make the Near West Side a healthier community we need _____?” and B, “To make the Near West Side a great place to live, work, play, and stay we need _____?” Both prompts were pilot tested with a group of 6 NWSP staff members who were also NWS residents. Prompt A generated a greater volume of responses that reflected actionable items than prompt B. Therefore, we used only focus prompt A in the subsequent steps of our project.

### GCM step 2

For the second step, idea generation, we invited adult community members to share their ideas for making NWS a healthier community. The goal of idea generation was to elicit a wide range of actionable ideas in response to focus prompt A, “To make the Near West Side a healthier community we need ____?” To maximize participation of community residents in idea generation, we added the focus prompt to the 2021 annual NWS resident survey. The resident survey is an electronic survey conducted door-to-door by the NWSP staff. The survey was also administered at community gatherings. Residents either responded orally and the NWSP representative entered their responses, or residents used the representative’s hand-held device to complete the survey themselves. Some residents accessed and completed the survey on their own devices through a link provided by an NWSP representative.

Focus prompt A generated 317 responses. First, we reviewed the responses and eliminated duplicate ideas. The remaining responses were synthesized into a list of ideas by editing for clarity, dividing responses that contained more than one idea into unique ideas, and grouping similar ideas into one idea. A list of 71 unique ideas was shared with NWSP’s executive director and associate director for review to ensure that all original ideas from the community were represented. The NWSP staff agreed that the 71 items were reflective of the ideas brought forth in the idea generation step. The final list included 71 discrete actionable ideas that were then used in the remaining GCM steps.

### GCM step 3

**Sorting and rating.** To recruit residents to participate in sorting and rating, flyers were distributed as door hangers and posted at neighborhood businesses, a public housing facility, and on the NWSP Facebook page. Sorting and rating took place in person at 2 readily accessible community sites. One session was held at a public housing facility dedicated to adults who are older and disabled, and multiple sessions were held over 2 weeks at a locally owned restaurant. Customers who came into the restaurant were also invited to participate. Volunteers, including Marquette University faculty, staff, and students and NWSP staff, sat with residents while they completed sorting and rating to provide instructions, to be available for questions, and to read ideas aloud for those who preferred that. As a token of appreciation, a $15 gift card, the cost of a complete meal at the restaurant, was provided to each person who completed the sorting and rating activity.

The sorting and rating activity was performed manually by each participant. For sorting, participants received a set of 71 cards. Each card was printed with 1 idea from the final list of ideas. Participants sorted the ideas into groups based on their interpretation of how the ideas were related. Participants were instructed to create more than 1 group and to place each card in only 1 group. After sorting all ideas into groups, participants provided names for their groups. Each person’s stacks of grouped cards with group name on the top card were paper-clipped together. Later, the name and ideas (identified by number) in each group were manually entered into The Concept System Global MAX software (Concept Systems Incorporated), a web-based platform for data management and analysis.

Rating of ideas followed sorting. Participants rated each idea based on 2 importance variables: 1) how important it was for making NWS a healthier community and 2) how important it was for making NWS a great place to live, work, play, and stay. Ratings were recorded on a 5-point Likert scale, ranging from 1, relatively unimportant, to 5, extremely important. The rating sheets were collected, and data were manually entered into The Concept System Global MAX software (Concept Systems Incorporated).

### GCM step 4

**Creating maps.** We used The Concept System Global MAX software to perform multidimensional scaling and hierarchical cluster analyses to generate cluster maps. A cluster map is a visual representation of how ideas are grouped or clustered together by participants. First, we created a point map by using a 2-dimensional multidimensional scaling analysis. On the point map, each point represented 1 idea, with distances between points determined by the frequency with which participants sorted the represented ideas into the same group. Ideas sorted together more frequently by more people resulted in smaller clusters. Next, using hierarchical cluster analysis, we partitioned the point maps into nonoverlapping clusters reflecting unique domains. This analytic approach produced many possible cluster solutions (ie, multiple maps with a varying number of clusters) from 1 point map. In the GCM methodology there is no predetermined appropriate number of clusters (18). Researchers, participants, and other invested parties collaboratively determined the ideal cluster solution (ie, number of clusters that resulted in conceptually meaningful and distinct domains).

Finally, by using the rating data, we generated a go-zone map to illustrate the prioritization of each idea based on the 2 importance variables — importance for a healthy community and importance for making NWS a great place to live, work, play, and stay. A go-zone chart is a bivariate plot with a point for each idea based on the average rating for the 2 importance variables. The go-zone chart comprises 4 quadrants made by dividing above or below the mean on the x-axis (healthy community) and the y-axis (live, work, play, and stay). Ideas in the upper-right quadrant represents those perceived to be important for a healthy community and for making NWS a great place to live, work, play, and stay.

### GCM step 5

**Interpreting maps.** During a final session, we invited NWS residents and staff from NWSP to meet as a group to review and provide feedback on the maps and their interpretations. First, we reviewed the point maps. We then reviewed the cluster maps we created. We presented attendees with the maps for clusters solutions ranging from 5 to 15 clusters (ie, in a 5-cluster solution, all 71 ideas were contained within 5 cluster groupings). The cluster map with 9 cluster groupings or domains was selected as the one that best represented participants’ priorities (Figure 1). We asked residents and staff members to read the ideas within each domain and to name that domain by suggesting a short phrase that best described the set of ideas. These suggested names guided the final domain names, which are high-quality and affordable housing, community-engaged public safety, health and wellness services, strong and inclusive neighborhoods, investments in young people, public infrastructure, sustainable businesses, alternative modes of transportation, and vibrant social spaces. We included individual ideas within each domain (Table). 

**Table:** Community-Driven Solutions, 71 Ideas Within Domains, Milwaukee, Wisconsin, 2016–2021

Domain	Ideas
Alternative modes of transportation	Opportunities for bicycles, fewer cars, high-speed train

Community-engaged public safety	Smoke detectors for homes, police that understand residents’ perspectives, police more involved with youth, newsletter from policing units, more police presence, less panhandling, barter system (trade skills and experience)

Health and wellness services	Safe spaces to work out, winter-time fitness options, access to mental health care, health clinic, drugstores, hospitals, farmers markets

High-quality and affordable housing	Higher wages, landlord involvement, owner-occupied homes, take down poorly run apartments, housing stability for renters, resources for homes repairs, affordable and high-quality housing, less homelessness

Investments in young people	Youth centers, childcare, children's recreation programs, playgrounds, summer camps for children

Public infrastructure	Address lead in water, enhanced bus services, neighborhood rideshare program, more street signs, lights at night, control reckless driving, street cameras and doorbell cameras, parking, street repair, trash cans and frequent trash collection

Strong and inclusive neighborhoods	Information about community services, intergenerational programs, people to help elderly, marketing benefits of living in NWS, place to meet with others from NWS, stronger sense of community, community events, support of neighborhood schools, support for neighborhood associations

Sustainable businesses	Opportunities for people of color (Black) to build businesses, more people employed, communication between residents and elected officials, community investments by the city, stronger sense of civic responsibility, communication among residents, funding to improve the area, investment in businesses, middle income families, support from the business community

Vibrant social spaces	Free fiber-optic internet installation, dog park, grocery store, a place for community, swimming pool, access to nutritious foods, coffee shops, community gardens, green spaces, health and wellness programs, restaurants

**Figure 2:**
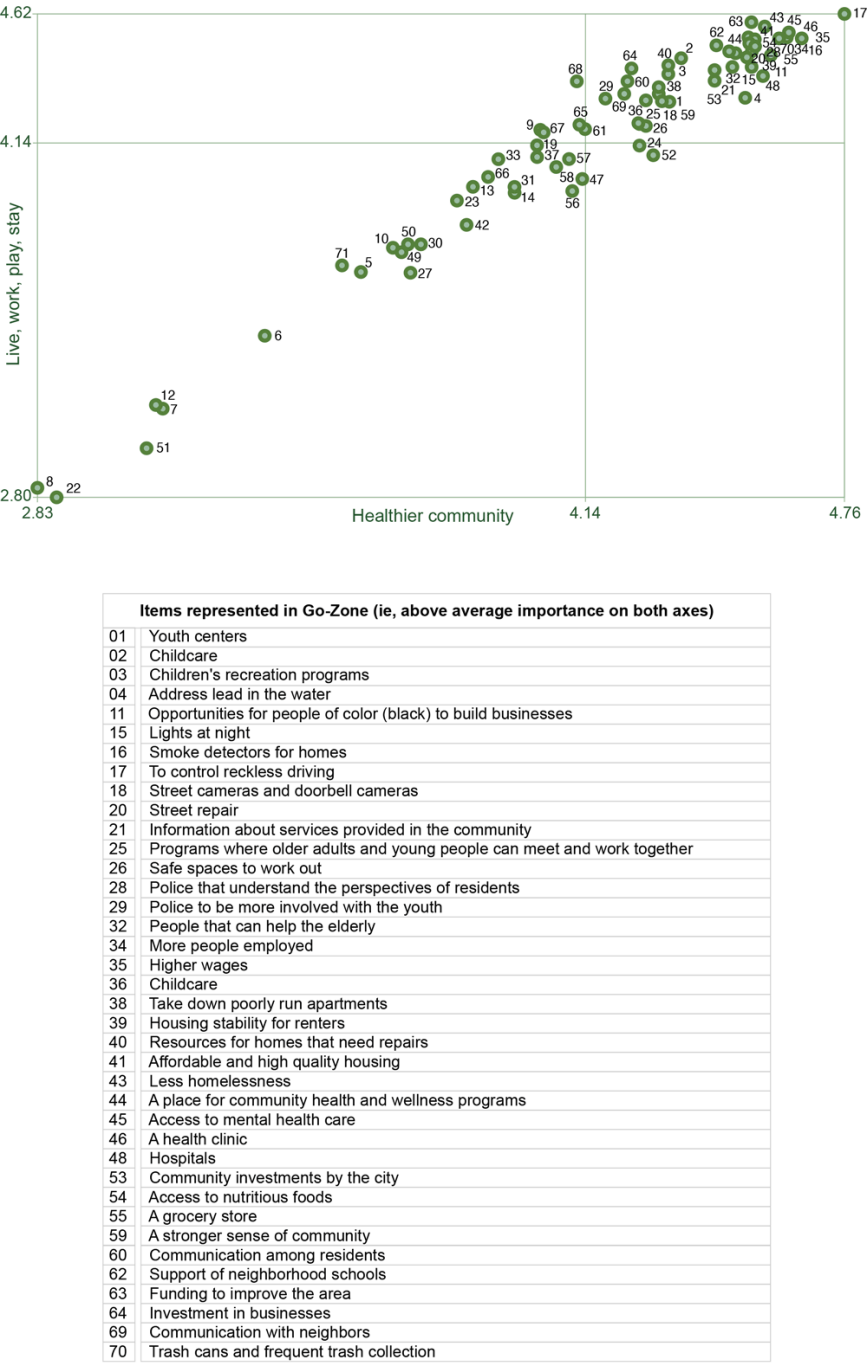
Go zone for Milwaukee’s Near West Side with points depicting average rating of importance for each idea. Four quadrants represent categories of** c**ommunity priorities based on 2 dimensions: "live, work, play, stay" (vertical axis) and "healthier community" (horizontal axis). Each quadrant represents a specific combination of high or low scores on these dimensions. On the vertical axis, the higher scores indicate greater importance within that dimension. On the horizontal axis, higher scores indicate greater importance. Ratings for importance for live, work, play stay (y-axis) range from 2.80 to 4.62 (mean 4.14) on scale of 1 to 5. Ratings for importance for healthier community (x-axis) range from 2.83 to 4.76 (mean 4.14) on scale of 1 to 5. Pearson’s product moment correlation coefficient *r = *0.98.

Finally, we created a go-zone that consisted of ideas prioritized as important for a healthy community and important for making NWS a great place to live, work, play, and stay (Figure 2). Across domains, 38 ideas were identified as being of high importance for both goals. These ideas represented 8 of the 9 domains. The domain “Alternative modes of transportation” was not represented in the go-zone.

## Enacting Principles of Community Engagement

NWSP has been physically embedded in the NWS of Milwaukee for 10 years. The organization and community residents have established routine communication pathways, hold regular shared events, and engage in joint community improvement efforts. Through intentional engagement, a shared knowledge of the history and contexts of the community serves as a foundational community engagement principle necessary to build a relationship of mutuality and trust (2). Having this baseline relationship provided a firm foundation for implementing a project designed to work with community residents to realize meaningful, sustainable change and improvements.

The project team included Marquette University researchers, staff, and students; NWSP leaders; and NWSP ambassadors. NWSP ambassadors were a unique asset because they both lived in NWS and worked for NWSP. The project team used a collaborative approach to make several key decisions in the research process (eg, focus prompt selection, incentives for participants, location of activities, domain names). This approach to decision-making helped to strengthen relationships within the project team. As part of the project team, NWSP ambassadors took the lead in presenting the project to residents, orienting volunteers to help with data collection, assisting participants with completing the sorting and rating, and serving as contact persons for questions and concerns. Because of the ambassadors’ dual role as NWSP staff and NWS residents, this model of shared leadership fostered trust and reciprocity within the project team and between the project team and community residents.

Another key principle of community engagement is co-creation with shared governance and sustainability (2). A critical element of this project was the feedback loop to share our results with the residents and the academic community. An important aspect of sustainability was delivering results that showed that peoples’ recommendations were included. In this project, the domains identified through the GCM process (Figure 2) were recognized as the community's vision and priorities. NWSP used these priorities to determine the organizations that would be ideal occupants to engage in conversations about the opportunity to be a tenant or service provider in the resource center. The resource center (now named Concordia 27) aligns with the domain of vibrant social spaces. It houses a café and ample space for residents and other community members to connect, collaborate, and build community. In addition to NWSP, Concordia 27 is home to a community-based organization specializing in wellness and trauma-informed education, trainings, and services (health and wellness services domain) and another community-based organization that provides workforce training for people with intellectual disabilities and those who were previously incarcerated (sustainable business domain). Concordia 27 also provides a kitchen incubator space and floor space for emerging small businesses. The domain of high-quality and affordable housing is addressed through the inclusion of 33 housing units that will be available for rent by senior citizens and working families.

## Conclusion

Community engagement in research operates on a continuum (19). We have described how a community–academic partnership, guided by the principles of community engagement, incorporated the perspectives of a diverse group of community members into a shared view of a healthy community. The project demonstrated 2 types of participatory relationships described previously by Key et al (19). First, the relationship between NWSP and researchers from Marquette University College of Nursing exemplifies a community-driven, community-led relationship given that NWSP identified a need and led the project with support from the college. Second, during the GCM process, community residents contributed equally to decision-making related to idea generation, the number and names of the domains, and the prioritization of the community-generated items.

Our findings underscore the importance of the social determinants of health for achieving overall health and well-being (20). By using a collaborative process, we identified several focus areas for community health interventions and programs. This work adds to a growing body of literature demonstrating how community–academic partnerships can harness available resources to effect change and advance shared goals (8,21,22).

Our project was overtly strength- and asset-based. Participants were acknowledged as experts on their lives and the neighborhoods in which they lived. Valuing people’s strengths and recognizing that community members are assets conveys respect. The focus prompt used to brainstorm ideas was strength-based; we did not ask for a list of deficits, nor did we use language that identified NWS or the people who live there as deficient. The GCM process invited community members to identify and prioritize actionable ideas. People increase involvement in initiatives when they identify with issues that they consider important, feel that they have influence in the initiative, and can make meaningful contributions toward the solution. Moreover, we minimized barriers to participation in the GCM process by advertising in advance through flyers delivered to homes and postings in visible locations. The data collection locations were readily accessible. To recognize the value of participants’ contributions we provided a gift certificate for a meal at a locally owned restaurant (one of the data collection sites).

Although community–academic partnerships are an opportunity to share, pool, and increase assets, the financial resources needed for Concordia 27 were beyond the capacity of the partnership. While NWSP founding institutions played a vital role in advocating for public funding for the project, their financial contributions were limited to furnishing and equipping the building. The findings from the GCM process provided critical insights into local needs and priorities, helping to shape Concordia 27 and attract investors who aligned with the community-engaged approach. The intentional collaboration with the community bolstered Concordia 27’s case for support and facilitated securing the necessary funding. The Concordia 27 project was launched with significant investment ($2 million or greater) from the State of Wisconsin, the federal government, and Milwaukee County. Additional funding was secured through historic tax credits, owner equity, and lending.

Although this project was conducted in the context of a long-standing, institutionalized collaboration, implementing the principles of community engagement though structured and participatory methodologies such as GCM can support building or growing a community–academic partnership. The amount of time that a partnership has been in existence is an important consideration when co-creating action plans and priorities. To facilitate success, outcomes and deliverables should be scaled to appropriately reflect the characteristics of the partnership and the resources available.

Engaging with community is a process that requires preparation, training, and intentionality. Community engagement and CEnR can generate mutually beneficial solutions that are strength-based, relevant, and aligned with partners’ priorities (3). Collaborating with communities harnesses the knowledge and expertise that individuals have about their own lives, informing interventions for better acceptance, uptake, and efficacy (23). For those seeking to address public health challenges and health equity, community engagement and CEnR are critical elements in the public health toolkit.
